# Circulating and cerebrospinal fluid soluble TREM2 levels and risk of perioperative neurocognitive disorders in surgical patients: a systematic review and meta-analysis

**DOI:** 10.3389/fnagi.2026.1899745

**Published:** 2026-10-01

**Authors:** Haotian Wu, Yujie Wang, Zhifeng Gao, Huan Zhang

**Affiliations:** 1 Department of Anesthesiology, Beijing Tsinghua Changgung Hospital, Beijing, China; 2 Medical Data Science Center, Beijing Tsinghua Changgung Hospital, Beijing, China

**Keywords:** postoperative delirium, postoperative cognitive dysfunction, sTREM2, neuroinflammation, biomarker, meta-analysis

## Abstract

**Background:**

Perioperative neurocognitive disorders (PND), including postoperative delirium (POD) and cognitive dysfunction (POCD), are common surgical complications in older adults. Neuroinflammation from microglial activation is a key mechanism. Soluble TREM2 (sTREM2) is a blood/CSF biomarker reflecting microglial activity. Existing studies on sTREM2 and PND risk have been inconsistent. We conducted the first meta-analysis to quantify this association.

**Methods:**

We searched PubMed, Web of Science, Scopus, APA PsycInfo, Embase, the Cochrane Library (CENTRAL), and https://www.ClinicalTrials.gov, CNKI, and Wanfang Data up to August 2026 for observational studies reporting perioperative sTREM2 levels in surgical patients with/without PND. Primary outcome was standardized mean difference (SMD) in sTREM2 between PND cases and controls, using a random-effects model. Heterogeneity was assessed via *I*^2^. Subgroup and sensitivity analyses were performed. PROSPERO: CRD420261386903.

**Results:**

Eight studies (1,154 patients) were included; seven with quantitative data (1,072 patients) were meta-analyzed. Patients with PND had higher sTREM2 levels than controls (SMD = 0.44, 95% CI: 0.02 to 0.87, *p* = 0.040; *I*^2^ = 86.5%). Subgroup analyses showed a positive direction of effect in CSF (SMD = 0.61, 95% CI: −0.02 to 1.23) but not in peripheral blood (SMD = 0.18, 95% CI: −0.13 to 0.49). Leave-one-out sensitivity analysis showed that the pooled estimate became non-significant after excluding the largest study, warranting cautious interpretation. One additional study reported that postoperative CSF sTREM2 elevation independently predicted POCD (adjusted OR = 1.07, 95% CI: 1.01–1.13).

**Conclusion:**

Elevated perioperative sTREM2 concentrations in cerebrospinal fluid, but not in peripheral blood, showed a modest positive association with PND risk. sTREM2 represents a candidate biomarker for perioperative cognitive complications; however, the very low certainty of the underlying evidence precludes definitive conclusions. Large-scale standardized studies are needed to validate its clinical utility.

**Systematic review registration:**

The systematic review was prospectively registered in PROSPERO (Unique Identifier: CRD420261386903), https://www.crd.york.ac.uk/prospero/.

## Introduction

Postoperative delirium (POD) and postoperative cognitive dysfunction (POCD) are common, debilitating neurological complications following surgery, with older adult patients at the highest risk of occurrence (Inouye et al., 2014; Evered et al., 2018). As two key subtypes of perioperative neurocognitive disorders (PND), POD and POCD present distinct clinical features. POD manifests as acute, fluctuating deficits in attention, consciousness, and cognitive function within days after surgery, while POCD is defined by persistent, long-term cognitive impairment that may last for weeks to months postoperatively (Rundshagen, 2014). Clinically, POD incidence ranges from 10 to 60% across different surgical procedures, and up to 40% of elderly patients develop POCD at hospital discharge, with 10–15% experiencing sustained cognitive decline at the 3-month postoperative follow-up (Moller et al., 1998; Monk et al., 2008).

These perioperative cognitive complications contribute to prolonged hospital stays, increased healthcare costs, accelerated long-term cognitive deterioration, higher rates of institutionalization, and elevated postoperative mortality (Steinmetz et al., 2009; Davis et al., 2012). Despite their substantial clinical and public health burden, there are currently no effective targeted interventions for PND prevention and treatment (Devlin et al., 2018). This clinical gap largely stems from an incomplete understanding of PND pathogenesis and the lack of reliable, clinically applicable predictive biomarkers (Fournier et al., 2015).

Accumulating preclinical and clinical evidence indicates that neuroinflammation serves as a core driving factor for PND onset and progression (Alam et al., 2018; Safavynia and Goldstein, 2018). Surgical trauma and anesthetic exposure trigger systemic inflammatory responses, disrupt blood–brain barrier integrity, and induce excessive activation of microglia, the resident innate immune cells in the central nervous system (CNS) (Terrando et al., 2010; Fan et al., 2020). Overactivated microglia release abundant pro-inflammatory cytokines (including IL-1β, IL-6, and TNF-α) and reactive oxygen species, leading to synaptic damage, impaired.

neuroplasticity, and neuronal loss, particularly in the hippocampus, a brain region critical for learning and memory (Cibelli et al., 2010; Hovens et al., 2015). Preclinical studies have further shown that inhibiting microglial overactivationeffectively attenuates surgery-induced cognitive deficits, verifying the causal role of microglia-mediated neuroinflammation in PND pathogenesis (Feng et al., 2017).

Triggering receptor expressed on myeloid cells 2 (TREM2) is a transmembrane protein specifically expressed in central microglia, which regulates microglial phagocytosis, inflammatory secretion, cell survival, and neuroinflammatory signaling (Hickman et al., 2013; Colonna and Wang, 2016). The extracellular domain of TREM2 can be cleaved by endogenous proteases to generate soluble TREM2 (sTREM2), a detectable biomarker in human cerebrospinal fluid, serum, and plasma (Piccio et al., 2008; Wunderlich et al., 2013). Elevated sTREM2 levels are generally considered to reflect microglial activity and have been widely studied in neurodegenerative disorders; however, because TREM2 also contributes to homeostatic microglial functions such as phagocytosis and cell survival, elevated sTREM2 levels should not be automatically equated with detrimental microglial overactivation. Previous research has shown that increased CSF sTREM2 levels appear in the early symptomatic stage of Alzheimer’s disease and correlate closely with tau pathology and neuronal damage (Suárez-Calvet et al., 2016; Henjum et al., 2018). Given the shared neuroinflammatory mechanisms between chronic neurodegeneration and acute perioperative cognitive injury, sTREM2 has emerged as a novel candidate biomarker for predicting PND risk (Cunningham, 2013; Fong et al., 2019).

Several clinical observational studies have explored the association between perioperative sTREM2 levels and postoperative cognitive complications, yet study findings remain inconsistent. Multiple studies have reported elevated sTREM2 concentrations in patientswho developed POD or POCD after surgery (Henjum et al., 2018; Wang et al., 2022; Ruhnau et al., 2023; Li et al., 2019), while other studies found no significant intergroup difference (Lin et al., 2023; Zhang et al., 2018; Cheng, 2022). These discrepancies likely result from clinical and methodological heterogeneity across studies, including variations in surgical types, patient age and baseline cognitive status, sTREM2 sampling time points, specimen types, and PND diagnostic criteria. To date, no.

Systematic review or meta-analysis has quantitatively synthesized existing evidence to clarify the relationship between sTREM2 and PND.

Accordingly, this systematic review and meta-analysis was designed to address three key research objectives: (1) to quantitatively assess the association between perioperative CSF/serum sTREM2 levels and PND, POD, and POCD risk in surgical patients; (2) to identify potential sources of inter-study heterogeneity through stratified subgroup analyses; (3) to evaluate the clinical potential of sTREM2 as a prognostic biomarker for perioperative neurocognitive disorders.

## Methods

This systematic review and meta-analysis complied fully with the 2020 Preferred Reporting Items for Systematic Reviews and Meta-Analyses (PRISMA) guidelines (Page et al., 2021) and was prospectively registered with the International Prospective Register of Systematic Reviews (PROSPERO: CRD420261386903). All included primary studies were approved by local institutional ethics committees, and written informed consent was obtained from all participants in each original trial.

### Search strategy

We systematically searched nine mainstream electronic databases, including PubMed, Web of Science Core Collection, Scopus, APA PsycInfo (Ovid), Embase, the Cochrane Library (CENTRAL), https://www.ClinicalTrials.gov, CNKI, and Wanfang Data, covering all published literature from database establishment to August 2026. No language or publication date restrictions were applied. The search strategy was structured around three core themes: sTREM2/TREM2 biomarkers, perioperative neurocognitive adverse outcomes, and surgical/anesthetic exposure. Detailed search strings for each database are provided in Supplementary Table S1. In addition to these bibliographic databases, we searched grey literature, including Chinese master’s theses indexed in CNKI and Wanfang Data and registered trials in https://www.ClinicalTrials.gov, with no language restrictions. The core PubMed search formula was as follows: (TREM2[All Fields] OR sTREM2[All Fields] OR “soluble TREM2”[All Fields] OR “Triggering Receptor Expressed on Myeloid Cells-2”[All Fields]) AND (“Delirium”[MeSH] OR “Cognitive Dysfunction”[MeSH] OR “Postoperative Cognitive Complications”[MeSH] OR “Neurocognitive Disorders”[MeSH] OR delirium[All Fields] OR POD[All Fields] OR POCD[All Fields] OR PND[All Fields] OR “postoperative cognitive dysfunction”[All Fields] OR “delayed neurocognitive recovery”[All Fields] OR “cognitive”[All. Fields] OR “neurocognitive”[All Fields]) AND (surgery[All Fields] OR. postoperative[All Fields] OR perioperative[All Fields] OR anesthesia[All Fields] OR operation[All Fields] OR surgical[All Fields]). Corresponding optimized search terms were adapted for other databases, using Emtree terms in Embase and topic-based terms in Chinese databases. Additionally, we manually screened the reference lists of all included studies and relevant systematic reviews to retrieve potentially eligible literature.

### Eligibility criteria

Study inclusion and exclusion criteria were formulated based on the PICOS framework:

*Population*: Adult patients (≥18 years) undergoing surgical procedures under general or regional anesthesia.

*Exposure*: Perioperative measurement of soluble TREM2 levels in cerebrospinal fluid, serum, or plasma.

*Comparator*: Surgical patients without postoperative delirium, postoperative cognitive dysfunction, or other perioperative neurocognitive disorders.

*Outcomes:* Primary outcome: PND incidence (POD diagnosed via the Confusion Assessment Method or DSM-5 criteria; POCD defined by standardized neuropsychological test batteries with *Z*-score classification). Secondary outcome: differences in perioperative sTREM2 concentrations between PND cases and non-PND controls.

*Study design*: Eligible studies included prospective/retrospective cohort studies and nested case–control studies. We excluded randomized controlled trials, animal experiments, systematic reviews, editorials, letters, case reports, and conference abstracts due to insufficient original clinical data.

### Study selection

Two independent reviewers (HW and YW) screened all retrieved records by title and abstract according to the predefined criteria. Full-text evaluations were performed for all potentially eligible articles to confirm final inclusion. Disagreements were resolved through discussion or consultation with a senior reviewer (ZG or HZ). The entire screening process was documented in a PRISMA 2020 flow diagram (Figure 1).

**Figure 1:**
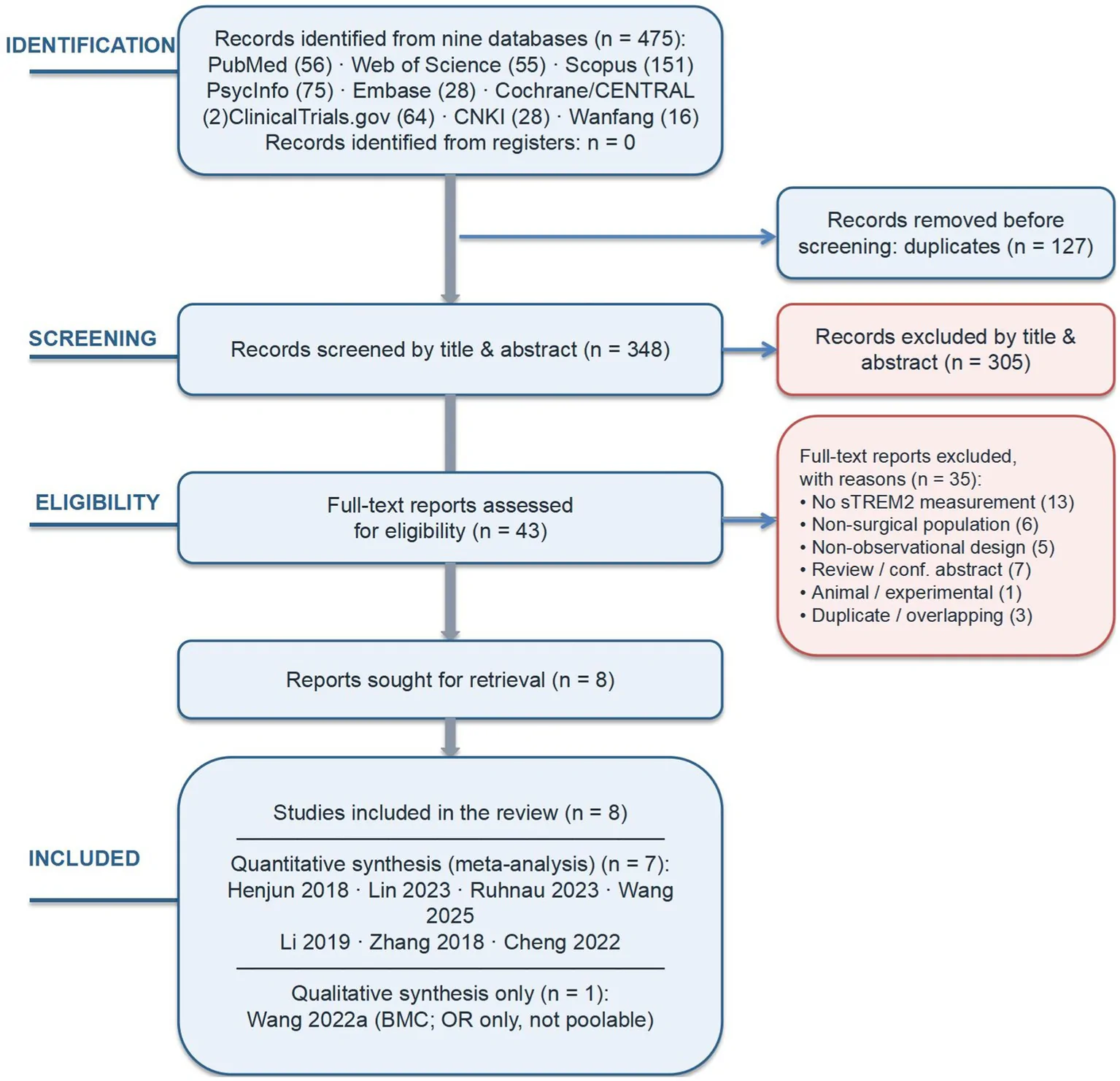
PRISMA 2020 flow diagram of study selection. A total of 475 records were identified from nine databases (PubMed, Web of Science, Scopus, APA PsycInfo, Embase, the Cochrane Library (CENTRAL), https://ClinicalTrials.gov, CNKI, and Wanfang Data). After removal of duplicates, 348 records were screened by title and abstract. Of these, 305 records were excluded. The full texts of the remaining 43 articles were assessed for eligibility. Thirty-five articles were excluded for the following reasons: no sTREM2 data (*n* = 13), non-surgical patients (*n* = 6), non-observational study design (*n* = 5), review/commentary (*n* = 7), animal study (*n* = 1), or duplicate/overlapping publication (*n* = 3). Finally, eight studies were included in the qualitative synthesis, of which seven provided complete descriptive statistics and were included in the quantitative meta-analysis.

### Data extraction

We used a standardized template to extract key data from eligible studies, including first author, publication year, study country, study design, surgical type, sample size (case and control subgroups), patient age and gender distribution, sTREM2 specimen type, sampling time points, cognitive assessment tools, and original sTREM2 quantitative data (mean ± standard deviation or median with interquartile range). For studies reporting only median and interquartile range values, we adopted validated conversion formulas to calculate corresponding mean and standard deviation (Wan et al., 2014). Studies reporting only adjusted odds ratios without raw quantitative data were included for qualitative analysis only. All extraction discrepancies were resolved by consensus. Baseline characteristics of included studies are summarized in Table 1. For each included study, we extracted the specific timing of sTREM2 sampling relative to surgery (e.g., preoperative baseline, immediate postoperative, or postoperative day 1–3). When available, we preferentially used the sTREM2 measurement obtained closest to the time of postoperative cognitive assessment for the primary pooled analysis. Studies that reported multiple postoperative time points were reviewed for consistency, but due to limited data, we were unable to perform separate meta-analyses by sampling window.

**Table 1:** Characteristics of included studies.

First author, year	Country	Surgery type	Sample size (case/control)	Age (years, mean or median)	sTREM2 source	Sampling time	Cognitive outcome	Main finding	Data format	Cognitive assessment tools
Henjum et al. (2018)	Norway	Hip fracture	15/44	85 (median)	CSF	Preoperative (at onset of spinal anesthesia)	Delirium (CAM)	Higher sTREM2 in POD among non-dementia subgroup	Median (IQR) converted to Mean ± SD	
Lin et al. (2023)	China	Knee/hip arthroplasty	92/92	66.5 (55.5–73) / 65 (56–71)	CSF	Preoperative	POD (CAM + MDAS)	No significant difference between groups	Mean ± SD	
Ruhnau et al. (2023)	Germany	Spine surgery	19/25	75.7 ± 5.8/75.6 ± 5.1	Serum	Preoperative	POD (DSM-5)	Preoperative sTREM2 predicts POD	Mean ± SD	
Wang et al. (2025)	China	Knee/hip arthroplasty	122/423	74 (IQR 8) / 59 (IQR 11)	CSF	Preoperative	POD (CAM)	High sTREM2 is independent risk factor for POD	Median (IQR) converted to Mean ± SD	
Wang (2022)^*^	China	Type A aortic dissection (Sun’s procedure)	19/29	59.5 ± 6.7/44.3 ± 8.7	CSF	Postoperative day 3	POCD (*Z*-score)	Postoperative CSF sTREM2 elevation associated with POCD (adjusted OR 1.07, 95% CI 1.01–1.13)	Adjusted OR only (narrative)	
Li et al. (2019)	China	Total knee arthroplasty	19/61	72 ± 6/71 ± 6	CSF	Preoperative (day 1)	POCD (*Z*-score, day 7)	Higher CSF and plasma sTREM2 in POCD (*p* < 0.05)	Mean ± SD	MMSE, visual verbal learning test, digit span (forward/backward), Stroop, digit-symbol substitution, trail making test
Cheng (2022)	China	Noncardiac surgery (general anesthesia)	21/59	84.2 ± 4.1/83.9 ± 3.2	Plasma	Postoperative (immediately after surgery)	POCD (day 7)	No significant association between plasma sTREM2 and POCD	Median (IQR) converted to Mean ± SD	MMSE, 3D-CAM, TICS-m (1-year follow-up)
Zhang et al. (2018)	China	Total knee arthroplasty	18/62	70 ± 6/70 ± 6	Plasma	Preoperative (T0) and postoperative (24 h, 72 h)	POCD (*Z*-score, day 7)	No significant difference in plasma TREM2 between groups	Mean ± SD	MMSE, visual verbal learning test, digit span, Stroop, digit-symbol substitution, trail making test

^*^The control group in Wang (2022) was significantly younger than the POCD group; this age discrepancy may confound the observed association.

POD, postoperative delirium; POCD, postoperative cognitive dysfunction; CSF, cerebrospinal fluid; CAM, Confusion Assessment Method; MDAS, Memorial Delirium Assessment Scale; DSM-5, Diagnostic and Statistical Manual of Mental Disorders, Fifth Edition; IQR, interquartile range; SD, standard deviation; OR, odds ratio.

### Risk of bias assessment

Two independent researchers assessed the methodological quality and risk of bias of all included observational studies using the Newcastle-Ottawa Scale (NOS) (Stang, 2010). The NOS evaluates study quality across three domains: group selection (0–4 stars), intergroup comparability (0–2 stars), and outcome assessment (0–3 stars).

Studies with a total NOS score ≥8 were defined as high-quality research. All assessment inconsistencies were resolved through discussion to ensure accuracy. Detailed quality evaluation results are presented in Table 2.

**Table 2:** Risk of bias assessment (Newcastle-Ottawa Scale for cohort studies).

Study	Selection (0–4)	Comparability (0–2)	Outcome (0–3)	Total (0–9)	Quality rating
Henjum et al. (2018)	4	2	3	9	High
Lin et al. (2023)	4	2	3	9	High
Ruhnau et al. (2023)	4	2	3	9	High
Wang et al. (2025)	4	2	3	9	High
Wang (2022)	4	2	2	8	High
Li et al. (2019)	4	2	2	8	High
Cheng (2022)	4	2	2	8	High
Zhang et al. (2018)	4	2	2	8	High

### Statistical analysis

All statistical analyses were performed using R software (v4.5.1) with the official meta package. Hedges’ g corrected standardized mean difference (SMD) with 95% confidence intervals (CIs) was used as the primary effect size to minimize small-sample bias. A DerSimonian-Laird random-effects model was applied for all pooled analyses to account for inherent clinical and methodological heterogeneity across observational studies (DerSimonian and Laird, 1986).

Inter-study heterogeneity was assessed using Cochran’s Q test and *I*^2^ statistic, with heterogeneity categorized as low (*I*^2^ = 25%), moderate (*I*^2^ = 50%), and high (*I*^2^ = 75%) per established standards (Higgins et al., 2003). Given the limited number of included studies (<10), formal quantitative publication bias testing was not feasible; a funnel plot was generated for qualitative visual evaluation. The certainty of evidence was assessed using the Grading of Recommendations Assessment, Development and Evaluation (GRADE) framework, and the overall certainty for the primary outcome was rated as very low, owing to serious inconsistency (*I*^2^ = 86.5%) and imprecision (a wide 95% confidence interval). A structured GRADE Summary of Findings is provided in Supplementary Table S5.

Because POD and POCD differ in clinical phenotype, timing, and diagnostic assessment, we prespecified stratified analyses by PND subtype in addition to the combined analysis; the decision to pool POD and POCD in the primary analysis was based on their shared neuroinflammatory substrate and the limited number of studies available for each outcome. Predefined subgroup analyses were therefore conducted by specimen type (CSF vs. peripheral blood) and PND subtype (POD vs. POCD). Quantitative data were available for both the POD and POCD subgroups, and results of the predefined subgroup analysis by PND subtype are reported in the Results. A fixed-effect model was additionally fitted as a sensitivity analysis to the random-effects model. Leave-one-out sensitivity analysis was performed by sequentially excluding individual studies to test the robustness of the pooled results. All statistical tests were two-tailed, and *p* < 0.05 was considered statistically significant.

## Results

### Study selection and baseline characteristics

A total of 475 literature records were retrieved from nine databases. After removal of 127 duplicate records, 348 records were screened by title and abstract, of which 305 were excluded. The full texts of the remaining 43 articles were assessed for eligibility. Ultimately, eight observational studies involving 1,154 surgical patients met the inclusion criteria. Seven studies with complete quantitative data (1,072 patients) were included in the final meta-analysis, and one study with only adjusted OR data was included for qualitative synthesis. The detailed screening process is shown in the PRISMA flow diagram (Figure 1).

Included studies were conducted in Norway, China, and Germany, covering multiple surgical procedures including hip fracture surgery, joint arthroplasty, spinal surgery, and thoracoabdominal aortic surgery. Most studies measured sTREM2 in CSF, while three studies used peripheral blood specimens. The baseline characteristics of each included study are summarized in Table 1. All studies adopted validated tools for POD and POCD diagnosis; the specific diagnostic instruments and the timing of cognitive assessments for each study are summarized in Table 1. All included studies achieved an NOS score ≥8, indicating high methodological quality and low risk of bias (Table 2).

### Pooled association between sTREM2 levels and PND risk

Quantitative pooling of seven eligible studies showed that surgical patients with postoperative neurocognitive disorders had higher sTREM2 concentrations than non-impaired surgical controls. The pooled random-effects SMD was 0.44 (95% CI: 0.02 to 0.87, *p* = 0.040), with substantial inter-study heterogeneity (*I*^2^ = 86.5%, *τ*^2^ = 0.27); the fixed-effect SMD was 0.55 (95% CI: 0.41 to 0.68, *p* < 0.001). The corresponding forest plot is presented in Figure 2.

**Figure 2:**
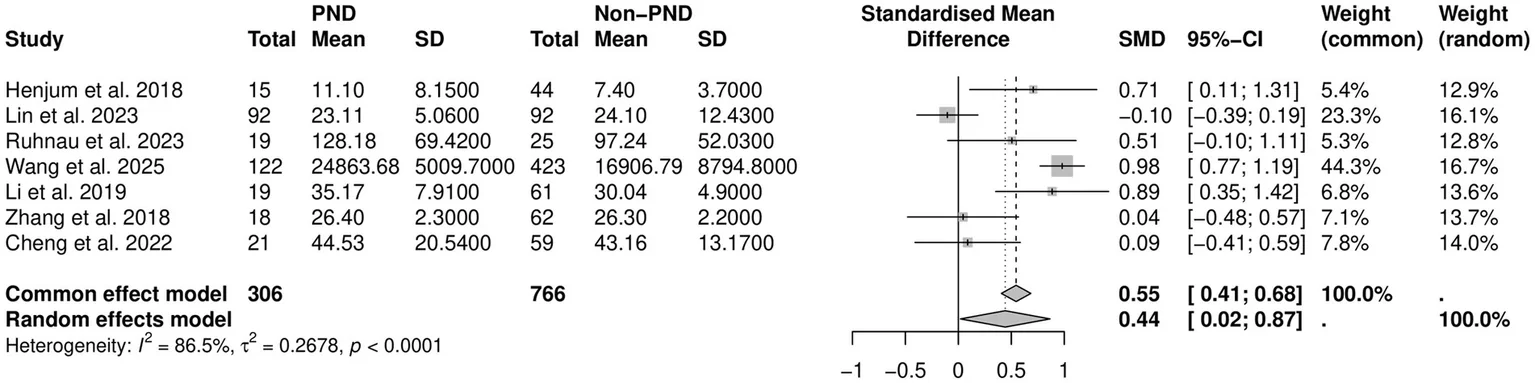
Forest plot of the association between sTREM2 levels and perioperative neurocognitive disorders (PND/POD/POCD). Standardized mean differences (SMD) with 95% confidence intervals (CIs) are shown for each study. The size of each square is proportional to the study weight in the random-effects model. The diamond represents the pooled SMD. The vertical dashed line indicates the null effect (SMD = 0). A random-effects model (DerSimonian-Laird) was used due to anticipated heterogeneity. The pooled analysis of seven studies (Henjum et al., 2018; Li et al., 2019; Lin et al., 2023; Ruhnau et al., 2023; Wang et al., 2025; Zhang et al., 2018; Cheng, 2022) showed that patients with PND had higher sTREM2 levels than controls (SMD = 0.44, 95% CI: 0.02 to 0.87, *p* = 0.040). Substantial heterogeneity was observed (*I*^2^ = 86.5%).

### Stratified subgroup analysis by specimen type

Subgroup analyses by biofluid type showed a similar direction of effect across specimens. The pooled SMD for CSF-based studies (*n* = 4) was 0.61 (95% CI: −0.02 to 1.23), with substantial within-subgroup heterogeneity (*I*^2^ = 91.8%). The pooled SMD for peripheral blood-based studies (*n* = 3) was 0.18 (95% CI: −0.13 to 0.49), with no within-subgroup heterogeneity (*I*^2^ = 0.0%). No statistically significant difference was detected between subgroups (*p* = 0.23); however, the peripheral blood estimate was derived from three relatively small studies and requires independent confirmation. An exploratory three-level breakdown by individual biofluid (matching Figure 3) showed that the three peripheral blood studies used two different matrices: serum in Ruhnau et al. (2023; *k* = 1) and plasma in Zhang et al. (2018) and Cheng (2022; *k* = 2), yielding a serum subgroup SMD of 0.51 (95% CI: −0.10 to 1.11) and a plasma subgroup SMD of 0.07 (95% CI: −0.29 to 0.43; *I*^2^ = 0.0%); the serum-specific estimate is therefore based on a single study and the plasma estimate on two small studies, and neither should be interpreted independently of the combined peripheral blood analysis. The subgroup forest plot is shown in Figure 3, with detailed data listed in Supplementary Table S3.

**Figure 3:**
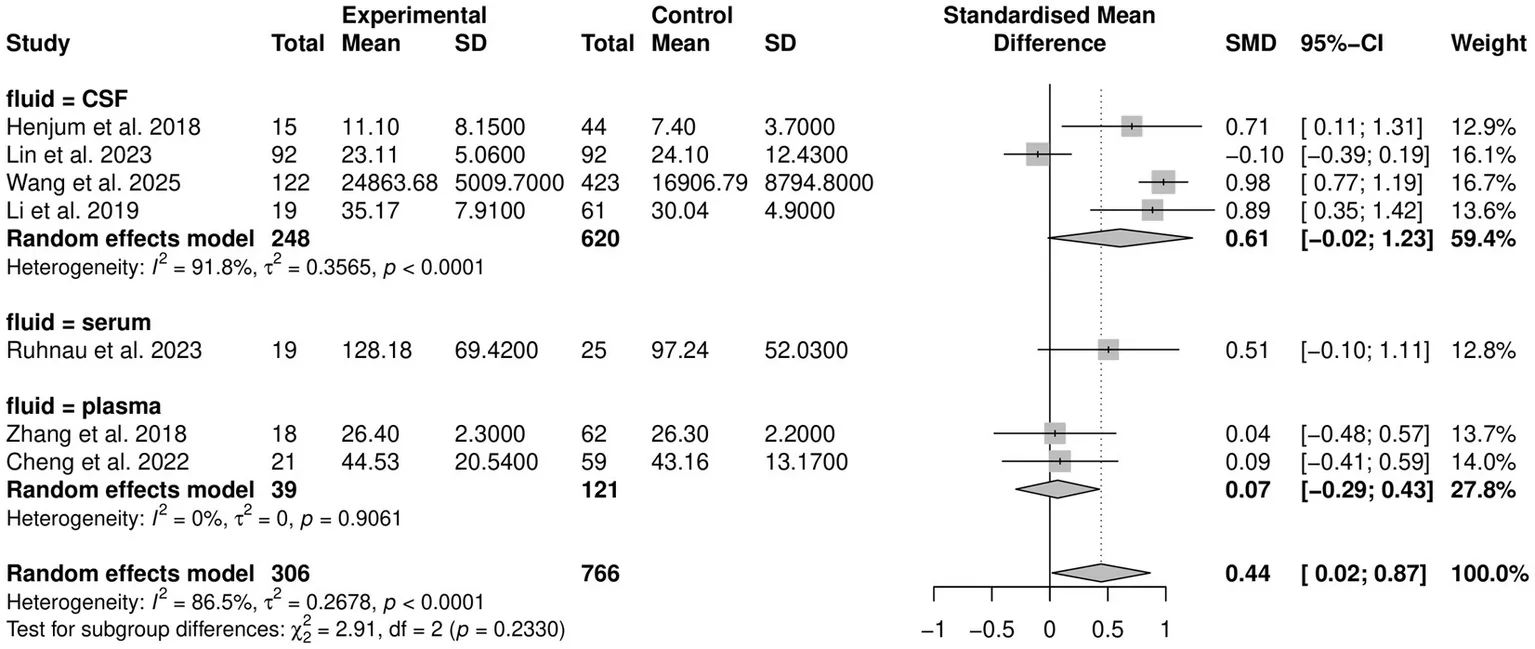
Subgroup forest plot by biofluid compartment (CSF, serum, and plasma). Subgroup analysis stratified by sTREM2 source. The pooled SMD for cerebrospinal fluid (CSF) studies (*n* = 4) was 0.61 (95% CI: −0.02 to 1.23), and the pooled SMD for peripheral blood studies (*n* = 3; Ruhnau et al., 2023; Zhang et al., 2018; Cheng, 2022) was 0.18 (95% CI: −0.13 to 0.49). The test for subgroup difference was not statistically significant (*p* = 0.23). The diamond for the CSF subgroup represents the pooled effect estimate from the random-effects model. The diamonds for each subgroup represent the pooled random-effects estimates within that subgroup.

### Stratified subgroup analysis by PND subtype

Subgroup analyses by PND subtype showed a consistent direction of effect across outcome definitions. The pooled SMD for POD studies (*n* = 4) was 0.52 (95% CI: −0.11 to 1.15), with substantial within-subgroup heterogeneity (*I*^2^ = 91.6%), and the pooled SMD for POCD studies (*n* = 3) was 0.33 (95% CI: −0.19 to 0.86, *I*^2^ = 67.8%). The between-subgroup difference was not statistically significant (*p* = 0.66), suggesting that the sTREM2-PND association was not driven by a specific outcome definition. Detailed subgroup statistics by PND subtype are provided in Supplementary Table S4. Although POD and POCD differ in clinical phenotype, timing, and diagnostic assessment, the consistent direction of effect across the two outcome definitions supports the decision to combine them in the primary analysis; nevertheless, the wide confidence intervals of both subgroup estimates indicate that these comparisons remain imprecise and should be interpreted cautiously.

### Sensitivity analysis results

Leave-one-out sensitivity analysis revealed that the pooled estimate was sensitive to individual studies. Exclusion of the Lin et al. (2023) study, which reported a negative effect size (SMD = −0.10), reduced heterogeneity from 86.5 to 74.0% and increased the pooled SMD to 0.56 (95% CI: 0.19 to 0.93). Conversely, exclusion of the largest study (Wang et al., 2025) attenuated the pooled estimate to a non-significant value (SMD = 0.32, 95% CI: −0.03 to 0.66, *p* = 0.071), indicating that the overall association was substantially influenced by this study. In a further analysis restricted to the four studies reporting directly measured means and standard deviations (Li et al., 2019; Lin et al., 2023; Ruhnau et al., 2023; Zhang et al., 2018; i.e., excluding median/IQR-derived estimates), the pooled SMD was 0.30 (95% CI: −0.17 to 0.77; *I*^2^ = 74.4%), further indicating the fragility of the pooled association. In addition, when the plasma arm of the study by Li et al. (2019) was pooled within the peripheral blood subgroup rather than the CSF subgroup, the peripheral blood estimate increased to 0.66 (95% CI: 0.09 to 1.22, *p* = 0.023), albeit with substantial heterogeneity (*I*^2^ = 92.3%). Exclusion of the remaining studies did not materially change the results. Detailed sensitivity analysis data are shown in Table 3.

**Table 3:** Sensitivity analysis (leave-one-out).

Study excluded	Pooled SMD (95% CI)	*I*^2^ (%)	Compared to full inclusion	Conclusion
None (full inclusion)	0.44 (0.02–0.87)	86.5	—	Base model
Henjum et al. (2018)	0.40 (−0.07–0.88)	88.7	Estimate attenuated, non-significant	Estimate attenuated, non-significant
Lin et al. (2023)	0.56 (0.19–0.93)	74.0	Heterogeneity reduced (*I*^2^ = 74.0%); estimate significant and higher	Lin is source of heterogeneity
Ruhnau et al. (2023)	0.43 (−0.04–0.91)	88.8	Estimate similar, non-significant	Estimate similar, non-significant
Wang et al. (2025)	0.32 (−0.03–0.66)	66.3	Estimate attenuated, non-significant	Estimate markedly attenuated, non-significant
Li et al. (2019)	0.37 (−0.10–0.85)	88.3	Estimate attenuated, non-significant (*p* = 0.125)	Estimate attenuated, non-significant
Zhang et al. (2018)	0.51 (0.04–0.97)	87.7	Estimate similar, significant (*p* = 0.033)	Estimate similar, significant
Cheng (2022)	0.50 (0.03–0.97)	87.8	Estimate similar, significant (*p* = 0.037)	Estimate similar, significant

SMD, standardized mean difference; CI, confidence interval.

### Publication bias assessment

Due to the small number of studies included in quantitative analysis (*n* = 7), formal statistical publication bias testing was not performed per meta-analysis guidelines. Visual inspection of the funnel plot revealed no obvious asymmetry, suggesting a low risk of publication bias, though this conclusion requires cautious interpretation given the limited sample size (Supplementary Figure S2).

### Qualitative supporting evidence

One eligible study reported that postoperative CSF sTREM2 elevation independently predicted POCD occurrence, with an adjusted OR of 1.07 (95% CI: 1.01–1.13, *p* = 0.022). This independent clinical evidence is consistent with the positive direction of association between elevated sTREM2 levels and PND observed in our quantitative meta-analysis (Supplementary Figure S1).

The thesis version of this cohort (Wang, 2022; registration ChiCTR2000038634) provided convergent findings with additional descriptive detail: on postoperative day 3, both CSF sTREM2 (*p* < 0.001) and serum sTREM2 (*p* = 0.004) levels were significantly higher in patients who developed POCD, and multivariable logistic regression confirmed that elevated postoperative CSF sTREM2 remained an independent risk factor for POCD (OR = 1.07, 95% CI: 1.01–1.13, *p* = 0.022). Because this gray-literature source reported no group-level summary statistics suitable for pooling and substantially overlapped with the published report, it was not counted as a separate study in the quantitative synthesis.

## Discussion

To our knowledge, this is the first meta-analysis to quantitatively clarify the association between sTREM2 levels and PND. Seven studies comprising 1,072 patients showed a positive association between elevated perioperative sTREM2 levels and PND (SMD = 0.44, 95% CI: 0.02 to 0.87), although the certainty of evidence was rated as very low owing to substantial heterogeneity and imprecision. Elevated CSF sTREM2 levels were significantly associated with PND risk, whereas the association for peripheral blood sTREM2 was positive but statistically non-significant.

The findings are hypothesis-generating with respect to the neuroinflammatory pathogenesis of PND and highlight the need for further research before sTREM2 can be considered a prognostic biomarker.

Microglial overactivation and subsequent neuroinflammation are widely recognized as the central pathological mechanism of PND (Alam et al., 2018; Safavynia and Goldstein, 2018). Surgical trauma and anesthetic stimulation trigger systemic inflammatory responses, disrupt blood–brain barrier integrity, and induce excessive microglial activation in the central nervous system. Activated microglia release large amounts of pro-inflammatory mediators, causing synaptic dysfunction and neuronal injury in cognition-related brain regions (Terrando et al., 2010; Fan et al., 2020). As a microglia-specific transmembrane receptor, TREM2 regulates multiple neuroinflammatory processes; its cleaved soluble fragment sTREM2 is considered a marker of microglial activity in the CNS (Piccio et al., 2008; Wunderlich et al., 2013); however, because TREM2 also mediates homeostatic microglial functions, the biological meaning of elevated perioperative sTREM2 should be interpreted cautiously. While previous research has confirmed the diagnostic and predictive value of sTREM2 in chronic neurodegenerative diseases, our study extends its application to acute perioperative neuroinflammatory brain injury.

The substantial heterogeneity detected in our overall analysis was partly attributable to the study by Lin et al., as its exclusion reduced the *I*^2^ value from 86.5 to 74.0% and increased the pooled SMD to 0.56 (95% CI: 0.19 to 0.93). The divergent findings of this study can be explained by notable clinical and methodological differences from other included research. First, this study enrolled a relatively young patient cohort (median age = 65 years), whereas other studies focused on elderly patients with age-related baseline microglial priming and chronic low-grade neuroinflammation (Norden and Godbout, 2013). Second, participants in this study had lower ASA physical status grades (I–II) and better preoperative physical function, with lower baseline inflammatory levels compared to the frailer patient populations in other studies. Third, variations in surgical stress intensity (elective joint arthroplasty vs. emergency hip fracture or complex aortic surgery) and anesthetic management further contributed to heterogeneous neuroinflammatory responses across studies. Exclusion of this outlier study reduced heterogeneity and strengthened the pooled effect, further supporting a positive association between sTREM2 elevation and PND risk.

Due to the limited number of included studies (*n* = 7), formal meta-regression or subgroup analyses based on clinical covariates (e.g., age, surgical type, sampling timing) were not feasible. Instead, we explored potential sources of heterogeneity through leave-one-out sensitivity analysis, as described above. Regarding the subgroup analysis stratified by biofluid type, the pooled estimate for the peripheral blood subgroup was derived from three studies (Ruhnau et al., 2023; Zhang et al., 2018; Cheng, 2022) and showed a positive but non-significant trend (SMD = 0.18, 95% CI: −0.13 to 0.49) with no within-subgroup heterogeneity (*I*^2^ = 0.0%), whereas the CSF subgroup showed a significant association (SMD = 0.61, 95% CI: −0.02 to 1.23). This divergence suggests that CSF sTREM2 may more directly reflect surgery-triggered central neuroinflammation and that peripheral blood sTREM2 cannot currently substitute for CSF measurement, although this finding should be interpreted with caution pending validation in larger, independent cohorts. Furthermore, sTREM2 sampling times varied across studies (preoperative baseline vs. postoperative days 1–3), and CSF and circulating sTREM2 reflect different biological compartments; the peripheral blood findings therefore require independent confirmation in prospective cohorts with standardized sampling protocols.

With respect to outcome definitions, we adopted the umbrella term perioperative neurocognitive disorders in line with the 2018 nomenclature update (Evered et al., 2018). None of the included studies reported delayed neurocognitive recovery (dNCR) as a distinct outcome; however, the cognitive assessment windows in several included studies (postoperative days 3–7 or before discharge) overlap with the dNCR window (≤30 days after surgery), which may have contributed to the observed heterogeneity. Importantly, the stratified subgroup analysis by PND subtype showed a consistent direction of effect for POD and POCD (between-subgroup *p* = 0.66), indicating that the pooled association was not driven by a specific outcome definition. Nevertheless, the overlap between early POCD and dNCR windows should be considered when interpreting the pooled estimates.

Compared with traditional inflammatory biomarkers (IL-6, IL-1β, CRP) that only reflect non-specific systemic inflammation, sTREM2 demonstrates higher central nervous system specificity and may reflect microglia-mediated central neuroinflammation (Filipello et al., 2022). Prior meta-analyses have confirmed elevated conventional inflammatory markers in PND patients, yet these markers cannot distinguish peripheral systemic inflammation from central neuroinflammatory injury. In contrast, sTREM2 has been proposed to reflect microglial-related processes with comparatively higher CNS specificity; however, it should not be regarded as a precise biomarker specific to neuroinflammation, given the pleiotropic homeostatic and phagocytic functions of TREM2. Multiple independent studies have reported that perioperative sTREM2 elevation was associated with POD and POCD, which may reflect both preoperative neuroinflammatory vulnerability and surgery-triggered microglial responses, although this interpretation requires prospective confirmation.

If prospectively validated, sTREM2 could have future clinical applications. First, perioperative sTREM2 measurement might eventually contribute to preoperative risk stratification; however, its current predictive value is not sufficient to guide clinical decisions, and prospective validation in independent cohorts is required before any such application can be considered. Second, the TREM2 signaling axis represents a potential, but not established, therapeutic target for PND: although preclinical studies suggest that TREM2 modulation may attenuate postoperative cognitive dysfunction (Han et al., 2022), TREM2-targeted interventions remain at an experimental stage, and their relevance to acute perioperative neurocognitive complications is uncertain.

Our study has several methodological strengths. First, this is the first meta-analysis specifically focusing on the sTREM2-PND correlation, with rigorous adherence to PRISMA 2020 guidelines and prospective PROSPERO registration. Second, all included studies were high-quality prospective cohort studies with NOS scores ≥8, ensuring low overall bias and reliable original data. Third, we conducted comprehensive subgroup and sensitivity analyses to explore heterogeneity sources and assess result stability, and adopted standardized methods to convert median and interquartile range data for pooled analysis (Wan et al., 2014).

Several limitations must be acknowledged. First, the small number of studies limited precision and precluded formal publication bias tests. Second, clinical heterogeneity (surgical types, sampling timing, assay methods) persisted despite random-effects models. Third, conversion of median/IQR data and use of different ELISA kits introduced measurement bias. Fourth, most studies were from Asian/European populations, limiting generalizability. Fifth, we could not adjust for potential confounders, including baseline cognitive status, frailty, APOE genotype, age, comorbidities, surgical type, and anesthetic or perioperative management, any of which could influence both sTREM2 levels and PND risk. Sixth, varied sampling times prevented time-stratified analysis. Seventh, although POD and POCD were analyzed separately in a prespecified subgroup analysis, they were combined in the primary analysis, and residual differences in outcome definition and cognitive assessment timing may have contributed to heterogeneity. Finally, no optimal clinical cutoff value has been established.

Future research should prioritize large-scale, multicenter prospective cohorts with standardized sTREM2 protocols and serial sampling. Combined measurement of multiple neuroinflammatory biomarkers may elucidate synergistic mechanisms. Interventional trials targeting the TREM2 pathway are warranted.

## Conclusion

In conclusion, this systematic review and meta-analysis suggests a modest but statistically significant association between elevated perioperative sTREM2 levels and PND, consistent with a role of neuroinflammation in PND pathogenesis; however, the association was small in magnitude, the evidence was rated as very low certainty, and the pooled estimate was sensitive to individual studies. sTREM2 should therefore be regarded as a candidate biomarker requiring prospective validation, rather than an established prognostic marker or therapeutic target.

Given the limitations of existing small-sample, heterogeneous studies, large-scale, standardized prospective clinical trials are urgently needed to further validate the clinical application value and unified diagnostic threshold of sTREM2 for PND prediction.

## Data Availability

The original contributions presented in the study are included in the article/Supplementary material, further inquiries can be directed to the corresponding authors.

## Supplementary material

SUPPLEMENTARY FIGURE S1 Forest plot of the independent cohort study by Wang et al. (2022). The odds ratio (OR) for postoperative CSF sTREM2 elevation predicting POCD was. 1.07 (95% CI: 1.01–1.13). The square size is proportional to the study weight. The diamond represents the pooled OR (not applicable for a single study).

SUPPLEMENTARY FIGURE S2 Funnel plot for publication bias. Each point represents an included study (*k* = 7). The vertical dashed line indicates the pooled random-effects estimate (SMD = 0.44). Visual inspection shows no obvious asymmetry. Formal statistical tests for publication bias were not performed due to the small number of studies (*k* < 10). Results should be interpreted with caution.

## References

[ref1] Alam A. Hana Z. Jin Z. Suen K. C. Ma D. (2018). Surgery, neuroinflammation and cognitive impairment. EBioMedicine 37, 547–556. doi: 10.1016/j.ebiom.2018.10.021, 30348620 PMC6284418

[ref2] Cheng S. (2022) *Risk factor analysis of postoperative cognitive dysfunction in elderly patients undergoing noncardiac surgery and its relationship with plasma strem2* (master's thesis), Soochow University Suzhou, China

[ref3] Cibelli M. Fidalgo A. R. Terrando N. Ma D. Monaco C. Feldmann M. . (2010). Role of interleukin-1beta in postoperative cognitive dysfunction. Ann. Neurol. 68, 360–368. doi: 10.1002/ana.22082 PMC4836445 20818791

[ref4] Colonna M. Wang Y. (2016). TREM2 variants: new keys to decipher Alzheimer disease pathogenesis. Nat. Rev. Neurosci. 17, 201–207. doi: 10.1038/nrn.2016.7, 26911435

[ref5] Cunningham C. (2013). Microglia and neurodegeneration: the role of systemic inflammation. Glia 61, 71–90. doi: 10.1002/glia.22350 22674585

[ref6] Davis D. H. J. Muniz Terrera G. Keage H. Rahkonen T. Oinas M. Matthews F. E. . (2012). Delirium is a strong risk factor for dementia in the oldest-old: a population-based cohort study. Brain 135, 2809–2816. doi: 10.1093/brain/aws190, 22879644 PMC3437024

[ref7] DerSimonian R. Laird N. (1986). Meta-analysis in clinical trials. Control. Clin. Trials 7, 177–188. doi: 10.1016/0197-2456(86)90046-2, 3802833

[ref8] Devlin J. W. Skrobik Y. Gélinas C. Needham D. M. Slooter A. J. C. Pandharipande P. P. . (2018). Clinical practice guidelines for the prevention and management of pain, agitation/sedation, delirium, immobility, and sleep disruption in adult patients in the ICU. Crit. Care Med. 46, e825–e873. doi: 10.1097/CCM.0000000000003299, 30113379

[ref9] Evered L. Silbert B. Knopman D. S. Scott D. A. DeKosky S. T. Rasmussen L. S. . (2018). Recommendations for the nomenclature of cognitive change associated with anaesthesia and surgery-2018. Br. J. Anaesth. 121, 1005–1012. doi: 10.1016/j.bja.2017.11.087, 30336844 PMC7069032

[ref10] Fan W. Mai L. Zhu X. Huang F. He H. (2020). The role of microglia in perioperative neurocognitive disorders. Front. Cell. Neurosci. 14:261. doi: 10.3389/fncel.2020.00261, 32973455 PMC7461842

[ref11] Feng X. Valdearcos M. Uchida Y. Lutrin D. Maze M. Koliwad S. K. (2017). Microglia mediate postoperative hippocampal inflammation and cognitive decline in mice. JCI Insight 2:e91229. doi: 10.1172/jci.insight.91229, 28405620 PMC5374063

[ref12] Filipello F. Goldsbury C. You S. F. Locca A. Karch C. M. Piccio L. (2022). Soluble TREM2: innocent bystander or active player in neurological diseases? Neurobiol. Dis. 165:105630. doi: 10.1016/j.nbd.2022.105630, 35041990 PMC10108835

[ref13] Fong T. G. Vasunilashorn S. M. Libermann T. Marcantonio E. R. Inouye S. K. (2019). Delirium and Alzheimer disease: a proposed model for shared pathophysiology. Int. J. Geriatr. Psychiatry 34, 781–789. doi: 10.1002/gps.5088, 30773695 PMC6830540

[ref14] Fournier A. Krause R. Winterer G. Schneider R. (2015). Biomarkers of postoperative delirium and cognitive dysfunction. Front. Aging Neurosci. 7:112. doi: 10.3389/fnagi.2015.00112 PMC4460425 26106326

[ref15] Han X. Cheng X. Xu J. Liu Y. Zhou J. Jiang L. . (2022). Activation of TREM2 attenuates neuroinflammation via PI3K/Akt signaling pathway to improve postoperative cognitive dysfunction in mice. Neuropharmacology 219:109231. doi: 10.1016/j.neuropharm.2022.109231 36041498

[ref16] Henjum K. Quist-Paulsen E. Zetterberg H. Blennow K. Nilsson L. N. G. Watne L. O. (2018). CSF sTREM2 in delirium—relation to Alzheimer’s disease CSF biomarkers Aβ42, t-tau and p-tau. J. Neuroinflammation 15, 304–319. doi: 10.1186/s12974-018-1331-1, 30390679 PMC6215363

[ref17] Hickman S. E. Kingery N. D. Ohsumi T. K. Borowsky M. L. Wang L. Means T. K. . (2013). The microglial sensome revealed by direct RNA sequencing. Nat. Neurosci. 16, 1896–1905. doi: 10.1038/nn.3554, 24162652 PMC3840123

[ref9001] Higgins J. P. T. Thompson S. G. Deeks J. J. Altman D. G. (2003). Measuring inconsistency in meta-analyses. BMJ 327, 557–560. doi: 10.1136/bmj.327.7414.557, 12958120 PMC192859

[ref18] Hovens I. B. van Leeuwen B. L. Nyakas C. Heineman E. van der Zee E. A. Schoemaker R. G. (2015). Postoperative cognitive dysfunction and microglial activation in associated brain regions in old rats. Neurobiol. Learn. Mem. 118, 74–79. doi: 10.1016/j.nlm.2014.11.009, 25460037

[ref19] Inouye S. K. Westendorp R. G. J. Saczynski J. S. (2014). Delirium in elderly people. Lancet 383, 911–922. doi: 10.1016/S0140-6736(13)60688-1, 23992774 PMC4120864

[ref20] Li C. Yu Y. Xie C. Wang B. Lin X. Dong R. . (2019). Predictive value of cerebrospinal fluid and plasma soluble triggering receptor expressed on myeloid cells levels for postoperative cognitive dysfunction in elderly patients. J. Precis. Med. 34, 499–505. doi: 10.13362/j.jpmed.201906008

[ref21] Lin Y. Yu N. Lin X. Deng X. Liu F. Tao H. . (2023). Preoperative cerebrospinal fluid biomarkers may be associated with postoperative delirium in patients undergoing knee/hip arthroplasty: the PNDABLE study. BMC Geriatr. 23:282. doi: 10.1186/s12877-023-03943-w, 37165310 PMC10173592

[ref22] Moller J. T. Cluitmans P. Rasmussen L. S. Houx P. Rasmussen H. Canet J. . (1998). Long-term postoperative cognitive dysfunction in the elderly ISPOCD1 study. ISPOCD investigators. International study of post-operative cognitive dysfunction. Lancet 351, 857–861. doi: 10.1016/s0140-6736(97)07382-0 9525362

[ref23] Monk T. G. Weldon B. C. Garvan C. W. Dede D. E. van der Aa M. T. Heilman K. M. . (2008). Predictors of cognitive dysfunction after major noncardiac surgery. Anesthesiology 108, 18–30. doi: 10.1097/01.anes.0000296071.19434.1e 18156878

[ref24] Norden D. M. Godbout J. P. (2013). Review: microglia of the aged brain: primed to be activated and resistant to regulation. Neuropathol. Appl. Neurobiol. 39, 19–34. doi: 10.1111/j.1365-2990.2012.01306.x, 23039106 PMC3553257

[ref25] Page M. J. McKenzie J. E. Bossuyt P. M. Boutron I. Hoffmann T. C. Mulrow C. D. . (2021). The PRISMA 2020 statement: an updated guideline for reporting systematic reviews. BMJ 372:n71. doi: 10.1136/bmj.n71, 33782057 PMC8005924

[ref26] Piccio L. Buonsanti C. Cella M. Tassi I. Schmidt R. E. Fenoglio C. . (2008). Identification of soluble TREM-2 in the cerebrospinal fluid and its association with multiple sclerosis and CNS inflammation. Brain 131, 3081–3091. doi: 10.1093/brain/awn217, 18790823 PMC2577803

[ref27] Ruhnau J. Müller J. Nowak S. Strack S. Sperlich D. Pohl A. . (2023). Serum biomarkers of a pro-neuroinflammatory state may define the pre-operative risk for postoperative delirium in spine surgery. Int. J. Mol. Sci. 24:10335. doi: 10.3390/ijms241210335, 37373482 PMC10298968

[ref28] Rundshagen I. (2014). Postoperative cognitive dysfunction. Dtsch. Arztebl. Int. 111, 119–125. doi: 10.3238/arztebl.2014.0119, 24622758 PMC3959222

[ref29] Safavynia S. A. Goldstein P. A. (2018). The role of neuroinflammation in postoperative cognitive dysfunction: moving from hypothesis to treatment. Front. Psych. 9:752. doi: 10.3389/fpsyt.2018.00752, 30705643 PMC6345198

[ref30] Stang A. (2010). Critical evaluation of the Newcastle-Ottawa scale for the assessment of the quality of nonrandomized studies in meta-analyses. Eur. J. Epidemiol. 25, 603–605. doi: 10.1007/s10654-010-9491-z, 20652370

[ref31] Steinmetz J. Christensen K. B. Lund T. Lohse N. Rasmussen L. S. ISPOCD Group (2009). Long-term consequences of postoperative cognitive dysfunction. Anesthesiology 110, 548–555. doi: 10.1097/ALN.0b013e318195b569, 19225398

[ref32] Suárez-Calvet M. Kleinberger G. Araque Caballero M. Á. Brendel M. Rominger A. Alcolea D. . (2016). sTREM2 cerebrospinal fluid levels are a potential biomarker for microglia activity in early-stage Alzheimer’s disease and associate with neuronal injury markers. EMBO Mol. Med. 8, 466–476. doi: 10.15252/emmm.201506123, 26941262 PMC5120370

[ref33] Terrando N. Monaco C. Ma D. Foxwell B. M. J. Feldmann M. Maze M. (2010). Tumor necrosis factor-α triggers a cytokine cascade yielding postoperative cognitive decline. Proc. Natl Acad. Sci. 2010;107:20518–22 10.1073/pnas.1014557107 PMC2996666 21041647

[ref34] Wan X. Wang W. Liu J. Tong T. (2014). Estimating the sample mean and standard deviation from the sample size, median, range and/or interquartile range. BMC Med. Res. Methodol. 14:135. doi: 10.1186/1471-2288-14-135, 25524443 PMC4383202

[ref35] Wang K. (2022) *The association between cerebrospinal fluid biomarker sTREM2 and postoperative cognitive dysfunction after cardiac surgery* (master's thesis), China Medical University Shenyang China

[ref36] Wang K. Cao X. Li Z. Liu S. Zhou Y. Guo L. . (2022). Anesthesia and surgery-induced elevation of CSF sTREM2 is associated with early cognitive dysfunction after thoracoabdominal aortic dissection surgery. BMC Anesthesiol. 22, 413–422. doi: 10.1186/s12871-022-01955-4, 36585610 PMC9805002

[ref37] Wang B. Wang X. Liang Y. Wang J. Hua S. Kong J. . (2025). Association of preoperative cerebrospinal fluid sTREM2 concentration with postoperative delirium and 3-year mortality in patients total knee or hip arthroplasty: a prospective cohort study. Int. J. Surg. 111, 9372–9383. doi: 10.1097/JS9.0000000000003465, 40928387 PMC12695191

[ref38] Wunderlich P. Glebov K. Kemmerling N. Tien N. T. Neumann H. Walter J. (2013). Sequential proteolytic processing of the triggering receptor expressed on myeloid cells-2 (TREM2) protein by ectodomain shedding and γ-secretase-dependent intramembranous cleavage. J. Biol. Chem. 288, 33027–33036. doi: 10.1074/jbc.M113.517540, 24078628 PMC3829152

[ref39] Zhang J. Wang B. Sun X. Wang M. Xu X. Bi Y. (2018). Relationship between triggering receptors expressed on myeloid cells and postoperative cognitive dysfunction in elderly patients. Chin. J. Anesthesiol. 38, 1421–1425. doi: 10.3760/cma.j.issn.0254-1416.2018.12.004

