# Supplementary material for: Circulating and cerebrospinal fluid soluble TREM2 levels and risk of perioperative neurocognitive disorders in surgical patients: a systematic review and meta-analysis
Source: Front Aging Neurosci. 2026 Oct 1;18:1899745. doi: 10.3389/fnagi.2026.1899745 (PMC13669114; doi:10.3389/fnagi.2026.1899745)
Supplement: Supplementary file 1 [file Data_Sheet_1.ZIP › 1899745_SupMaterial_updated/Table 4.DOCX]

## **Supplementary Table S1. Detailed search strategies for all databases**

| Database | Search strategy | Date searched | Records retrieved |
| --- | --- | --- | --- |
| PubMed | ("Triggering Receptor Expressed on Myeloid Cells-2"[Mesh] OR "Triggering Receptor Expressed on Myeloid Cells-2"[Supplementary Concept] OR TREM2[All Fields] OR sTREM2[All Fields] OR "soluble TREM2"[All Fields]) AND ("Delirium"[Mesh] OR "Cognitive Dysfunction"[Mesh] OR "Postoperative Cognitive Complications"[Mesh] OR "Neurocognitive Disorders"[Mesh] OR delirium[All Fields] OR POD[All Fields] OR POCD[All Fields] OR PND[All Fields] OR "delayed neurocognitive recovery"[All Fields] OR "perioperative neurocognitive disorder*"[All Fields] OR "cognitive dysfunction"[All Fields]) AND ("Surgical Procedures, Operative"[Mesh] OR "Anesthesia"[Mesh] OR "Perioperative Period"[Mesh] OR "Postoperative Period"[Mesh] OR surgery[All Fields] OR operation*[All Fields] OR postoperative[All Fields] OR perioperative[All Fields] OR anesthesia[All Fields] OR surgical[All Fields]) | August 2026 | 56 |
| Web of Science (Core Collection) | TS=(TREM2 OR sTREM2 OR "soluble TREM2") AND TS=(delirium OR "cognitive" OR "neurocognitive") AND TS=(surgery OR operation OR postoperative OR perioperative OR anesthesia) | August 2026 | 55 |
| Scopus | ( "TREM2" OR "sTREM2" OR "soluble TREM2" OR "triggering receptor expressed on myeloid cells 2" ) AND ( "postoperative delirium" OR "POD" OR "postoperative cognitive dysfunction" OR "POCD" OR "perioperative neurocognitive disorder*" OR "PND" OR "emergence delirium" ) AND ( "surgery" OR "operation" OR "postoperative" OR "perioperative" OR "anesthesia" OR "anaesthesia" ) | August 2026 | 151 |
| APA PsycInfo (Ovid) | exp *triggering receptor expressed on myeloid cells 2/ OR exp *sTREM2/ OR exp *TREM2 protein/ OR (sTREM2 OR "sTREM2" OR "soluble TREM2" OR TREM2).ti,ab,id,hw. [concept 1 only; combined with outcome and surgery terms using AND] | August 2026 | 75 |
| [ClinicalTrials.gov](https://clinicaltrials.gov/) | Condition/disease: postoperative delirium OR postoperative cognitive dysfunction OR perioperative neurocognitive disorders  Other terms: TREM2 OR sTREM2 OR soluble TREM2 Study type: Observational (Cohort or Case-Control) Age group: Adult (18+) | August 2026 | 64 |
| Embase (Embase.com) | ('triggering receptor expressed on myeloid cells 2'/exp OR 'trem2':ti,ab,kw OR 'strem2':ti,ab,kw OR 'soluble trem2':ti,ab,kw) AND ('delirium'/exp OR 'delayed neurocognitive recovery':ti,ab,kw OR 'dncr':ti,ab,kw OR 'postoperative cognitive dysfunction':ti,ab,kw OR 'pocd':ti,ab,kw OR 'postoperative delirium':ti,ab,kw OR 'pod':ti,ab,kw OR 'perioperative neurocognitive disorder*':ti,ab,kw OR 'pnd':ti,ab,kw) AND ('surgery'/exp OR 'operation'/exp OR 'postoperative period'/exp OR 'anesthesia'/exp OR 'perioperative':ti,ab,kw OR 'anaesthesia':ti,ab,kw) | August 2026 | 28 |
| Cochrane Library (CENTRAL) | #1 MeSH descriptor: [Triggering Receptor Expressed on Myeloid Cells 2] explode all trees  #2 (TREM2 OR sTREM2 OR "soluble TREM2" OR "triggering receptor expressed on myeloid cells 2"):ti,ab,kw  #3 MeSH descriptor: [Delirium] explode all trees  #4 (delirium OR POD OR POCD OR PND OR dNCR OR "delayed neurocognitive recovery" OR "postoperative cognitive dysfunction" OR (perioperative NEXT neurocognitive NEXT disorder*) OR "cognitive dysfunction"):ti,ab,kw  #5 MeSH descriptor: [Perioperative Period] explode all trees  #6 (surgery OR surgical OR operation* OR postoperative OR perioperative OR anesthesia OR anaesthesia):ti,ab,kw  #7 (#1 OR #2) AND (#3 OR #4) AND (#5 OR #6) | August 2026 | 2 |
| CNKI | SU=('TREM2'+'sTREM2'+'可溶性TREM2'+'髓系细胞触发受体2') AND SU=('谵妄'+'术后谵妄'+'认知功能障碍'+'术后认知功能障碍'+'围术期神经认知障碍'+'神经认知障碍') AND SU=('手术'+'麻醉'+'术后'+'围手术期'+'围术期') | August 2026 | 28 |
| Wanfang Data | 主题:("TREM2" OR "sTREM2" OR "可溶性TREM2" OR "髓系细胞触发受体2") AND 主题:("谵妄" OR "术后谵妄" OR "认知功能障碍" OR "术后认知功能障碍" OR "围术期神经认知障碍" OR "神经认知障碍") AND 主题:("手术" OR "麻醉" OR "术后" OR "围手术期" OR "围术期") | August 2026 | 16 |

Note: Embase, Cochrane Library, CNKI, and Wanfang Data were additionally searched in August 2026 during revision; the original five sources were searched in May 2026.
