# Supplementary material for: Circulating and cerebrospinal fluid soluble TREM2 levels and risk of perioperative neurocognitive disorders in surgical patients: a systematic review and meta-analysis
Source: Front Aging Neurosci. 2026 Oct 1;18:1899745. doi: 10.3389/fnagi.2026.1899745 (PMC13669114; doi:10.3389/fnagi.2026.1899745)
Supplement: Supplementary file 1 [file Data_Sheet_1.ZIP › 1899745_SupMaterial_updated/Table 5.DOCX]

**Supplementary Table S2. Data extraction for meta-analysis**

*Values as reported in each original publication are shown together with the values entered into the meta-analysis. Because absolute concentrations depend on the ELISA platform and unit, all pooled estimates used Hedges' g standardized mean differences (SMD).*

| Study (year) | Biofluid | Outcome | PND (n) | PND sTREM2 (as reported) | Non-PND (n) | Non-PND sTREM2 (as reported) | Unit | Values in meta-analysis (mean ± SD; PND / Non-PND) | Data extraction |
| --- | --- | --- | --- | --- | --- | --- | --- | --- | --- |
| Cerebrospinal fluid (CSF) | | | | | | | | | |
| Henjum et al. 2018 | CSF | POD | 15 | 11.1 (IQR 11.0) | 44 | 7.4 (IQR 5.0) | ng/mL | 11.1 ± 8.15 / 7.4 ± 3.70 | Median (IQR); mean approximated by median; SD = IQR/1.35 |
| Lin et al. 2023 | CSF | POD | 92 | 23.11 ± 5.06 | 92 | 24.10 ± 12.43 | ng/mL | 23.11 ± 5.06 / 24.10 ± 12.43 | Original Table 2 (mean ± SD) |
| Wang et al. 2025 | CSF | POD | 122 | 24,863.68 (IQR 6,762.07) | 423 | 16,906.79 (IQR 11,873.03) | pg/mL | 24,863.68 ± 5,009.7 / 16,906.79 ± 8,794.8 | Median (IQR); mean approximated by median; SD = IQR/1.35 |
| Li et al. 2019 | CSF | POCD | 19 | 35.17 ± 7.91 | 61 | 30.04 ± 4.90 | ng/L | 35.17 ± 7.91 / 30.04 ± 4.90 | Original (mean ± SD) |
| Peripheral blood | | | | | | | | | |
| Ruhnau et al. 2023 | Serum | POD | 19 | 128.18 ± 69.42 | 25 | 97.24 ± 52.03 | pg/mL | 128.18 ± 69.42 / 97.24 ± 52.03 | Original Table 2 (mean ± SD) |
| Zhang et al. 2018 | Plasma | POCD | 18 | 26.4 ± 2.3 (T0) | 62 | 26.3 ± 2.2 (T0) | ng/L | 26.4 ± 2.3 / 26.3 ± 2.2 | Original (mean ± SD), preoperative (T0) |
| Cheng et al. 2022 | Plasma | POCD | 21 | 47.85 [29.00, 56.73] | 59 | 41.45 [35.13, 52.91] | ng/mL | 44.53 ± 20.54 / 43.16 ± 13.17 | Median [Q1, Q3] (immediately after surgery); mean = (Q1+M+Q3)/3; SD = IQR/1.35 (Wan 2014) |
| Included descriptively (not pooled) | | | | | | | | | |
| Wang et al. 2022a | CSF | POCD | 19 | Not extractable (reported as OR) | 29 | — | — | — | Narrative only; no group-level concentrations reported. Postoperative CSF sTREM2 elevation associated with POCD: adjusted OR 1.07 (95% CI 1.01–1.13). |

a. Unit conversions: 1 ng/mL = 1 µg/L = 1000 pg/mL = 1000 ng/L. Absolute concentrations cannot be compared across studies because ELISA kits differ; all meta-analytic pooling used Hedges' g SMD.

b. Transformation methods (Wan et al. 2014, BMC Med Res Methodol 14:135): for studies reporting only median and IQR span (Henjum 2018; Wang 2025), the median was used to approximate the mean and SD was estimated as IQR/1.35 (normal approximation); for Cheng et al. 2022, which reported the full median [Q1, Q3] interval, the mean was estimated as (Q1 + M + Q3)/3 and SD as IQR/1.35. Studies reporting mean ± SD directly (Lin 2023; Ruhnau 2023; Li 2019; Zhang 2018) were used as reported.

c. Sampling time: Wang 2025 and Henjum 2018 – preoperative CSF; Zhang 2018 – preoperative (T0) plasma; Cheng 2022 – plasma sampled immediately after surgery; POCD assessed on postoperative day 7; Lin 2023 – preoperative CSF; sampling times for Li 2019 and Ruhnau 2023 are summarized in Table 1 of the manuscript.

d. Wang et al. 2025: the large absolute values reflect the pg/mL unit for CSF concentrations and are not a data-entry error. The IJS English version (n = 545; POD 122 / non-POD 423) was used; a separate Chinese cohort report (n = 519; POD 112 / non-POD 407) was not included.

e. Wang et al. 2022a (n = 48, POCD 19 / non-POCD 29) reported only an OR (adjusted 1.07, 95% CI 1.01–1.13) without group-level concentrations and was therefore summarized narratively.
