# Supplementary material for: Circulating and cerebrospinal fluid soluble TREM2 levels and risk of perioperative neurocognitive disorders in surgical patients: a systematic review and meta-analysis
Source: Front Aging Neurosci. 2026 Oct 1;18:1899745. doi: 10.3389/fnagi.2026.1899745 (PMC13669114; doi:10.3389/fnagi.2026.1899745)
Supplement: Supplementary file 1 [file Data_Sheet_1.ZIP › 1899745_SupMaterial_updated/Table 6.DOCX]

## **Supplementary Table S3. Subgroup analysis by sample type**

| Subgroup | Number of studies | Pooled SMD (95% CI) | I² (%) | P for subgroup difference |
| --- | --- | --- | --- | --- |
| CSF sTREM2 | 4 (Henjum, Lin, Wang 2025, Li) | 0.61 (−0.02 – 1.23) | 91.8 | 0.23 |
| Serum sTREM2 | 1 (Ruhnau) | 0.51 (−0.10 – 1.11) | — | 0.23 |
| Plasma sTREM2 | 2 (Zhang, Cheng) | 0.07 (−0.29 – 0.43) | 0.0 | 0.23 |

^*^Note: The substantial heterogeneity within the CSF subgroup (I² = 91.8%) may be attributed to differences in surgical types, patient age, and assay methods across studies.
