# Supplementary material for: Circulating and cerebrospinal fluid soluble TREM2 levels and risk of perioperative neurocognitive disorders in surgical patients: a systematic review and meta-analysis
Source: Front Aging Neurosci. 2026 Oct 1;18:1899745. doi: 10.3389/fnagi.2026.1899745 (PMC13669114; doi:10.3389/fnagi.2026.1899745)
Supplement: Supplementary file 1 [file Data_Sheet_1.ZIP › 1899745_SupMaterial_updated/Table 7.DOCX]

**Supplementary Table S4. Subgroup analysis by PND subtype (POD vs POCD).**

| Subgroup | Number of studies | Pooled SMD (95% CI) | I² (%) | P for subgroup difference |
| --- | --- | --- | --- | --- |
| POD | 4 (Henjum, Lin, Ruhnau, Wang 2025) | 0.52 (−0.11 – 1.15) | 91.6 | 0.66 |
| POCD | 3 (Li, Zhang, Cheng) | 0.33 (−0.19 – 0.86) | 67.8 | 0.66 |
