# Supplementary material for: Circulating and cerebrospinal fluid soluble TREM2 levels and risk of perioperative neurocognitive disorders in surgical patients: a systematic review and meta-analysis
Source: Front Aging Neurosci. 2026 Oct 1;18:1899745. doi: 10.3389/fnagi.2026.1899745 (PMC13669114; doi:10.3389/fnagi.2026.1899745)
Supplement: Supplementary file 1 [file Data_Sheet_1.ZIP › 1899745_SupMaterial_updated/Table 8.DOCX]

**Supplementary Table S5. GRADE Summary of Findings for the primary outcome.**

| Outcome | No. of studies (No. of participants) | Pooled effect (95% CI) | Certainty of evidence (GRADE) |
| --- | --- | --- | --- |
| Perioperative sTREM2 levels (PND vs non-PND) | 7 studies (1,072 participants; 306 PND events) | Random-effects SMD = 0.44 (0.02 to 0.87); fixed-effect SMD = 0.55 (0.41 to 0.68) | ○○○○ VERY LOWᵇ Downgraded for serious inconsistency (I² = 86.5%, τ² = 0.27) and serious imprecision (95% CI includes values near the null). Risk of bias, indirectness, and publication bias were not serious. |
| Subgroup analysis by sample type (CSF, serum, plasma) | CSF: 0.61 (−0.02–1.23); serum: 0.51 (−0.10–1.11); plasma: 0.07 (−0.29–0.43) | Between-subgroup P = 0.23 (non-significant) | ○○○○ VERY LOWᵇ for all subgroups |
